# Supplementary material for: Circulating miR-499a-5p Is a Potential Biomarker of MYH7—Associated Hypertrophic Cardiomyopathy
Source: Int J Mol Sci. 2022 Mar 30;23(7):3791. doi: 10.3390/ijms23073791 (PMC8998764; doi:10.3390/ijms23073791)
Supplement: Supplementary file 1 [file ijms-23-03791-s001.zip › ijms-1618015-supplementary.pdf]

International Journal of
Molecular Sciences

MDPI

Table S1. Circulating miRNAs, dysregulated in plasma of HCM patients compared to individuals of the control group.

№	MiRNA ID	LogFC	*p* value	FDR *p* value
		**Downregulated miRNAs in HCM**		
1	hsa-miR-208b	−3.17	0.00000005	1.35×10^{-5}
2	hsa-miR-499a-5p	−3.84	0.00032	0.043
3	hsa-miR-1255b	−3.52	0.0022	0.15
4	hsa-miR-454	−2.87	0.002	0.15
5	hsa-miR-339-3p	−2.62	0.0092	0.28
6	hsa-miR-1468	−2,58	0.029	0.28
7	hsa-miR-485-5p	−2.37	0.013	0.28
8	hsa-miR-320c	−2.32	0.029	0.28
9	hsa-miR-92a-1*	−2,26	0.017	0.28
10	hsa-miR-411	−2.25	0.029	0.28
11	hsa-miR-199a-5p	−2.25	0.029	0.28
12	hsa-miR-3163	−2.25	0.029	0.28
13	hsa-miR-99a	−2.13	0.029	0.28
14	hsa-miR-26b	−1.06	0.0075	0.28
15	hsa-let-7g	−1.02	0,0082	0.28
16	hsa-miR-144	−1	0.027	0.28
17	hsa-miR-199b-5p	−2.31	0.031	0.29
18	hsa-miR-34c-5p	−1.88	0.034	0.29
19	hsa-miR-450a	−1.88	0.034	0.29
20	hsa-miR-616*	−1.88	0.034	0,29
21	hsa-miR-2110	−1.88	0.039	0.31
22	hsa-miR-133a	−2.17	0.044	0.34
23	hsa-miR-203	−1.89	0.046	0.35
		**Upregulated miRNAs in HCM**		
1	hsa-miR-339-5p	2.9	0.012	0.28
2	hsa-miR-335*	2.75	0.013	0.28
3	hsa-let-7a*	2.42	0.022	0.28
4	hsa-miR-93*	2.4	0.028	0.28
5	hsa-miR-1270	2.31	0.028	0.28
6	hsa-miR-873	2.31	0.028	0.28
7	hsa-miR-214*	2.26	0.028	0.28

8	hsa-miR-210	1.94	0.023	0.28
9	hsa-miR-409-3p	1.64	0.019	0.28
10	hsa-miR-342-5p	1.62	0.025	0.28
11	hsa-miR-10b	1.03	0.0069	0.28
12	hsa-miR-486-5p	0.42	0.01	0.28
13	hsa-miR-758	2.04	0.039	0.31

*passenger strand of miRNA.

Table S2. General clinical characteristics of participants of the study according to genotyping status.

**Groups**	**Carriers of P/LP variants in *MYH7* gene (n=7)**	**Carriers of P/LP variants in *MYBPC3* gene (n=8)**	**Carriers of P/LP variants in other HCM-associated genes and genotype-negative HCM patients (n=14)**
Female, n (%)	4 (57)	3 (37.5)	5 (35.7)
Age, years	48 ± 15.2	43 ± 10.0	51 ± 13.3
BMI, kg/m2	28.14 ± 6.18	26.10 ± 3.55	26.81 ± 3.72
5-year HCM Risk-SCD (%)	3.69 ± 1.52	3.54 ± 2.14	4.19 ± 2.91
Family history of HCM, n (%)	3 (42.9)	6 (75)	3 (21.4)
Family history of SCD, n (%)	1 (14.3)	3 (37.5)	1 (7)
Atrial fibrillation, n (%)	1 (14.3)	1 (12.5)	2 (14.3)
Ventricular tachycardia, n (%)	1 (14.3)	1 (12.5)	4 (28.6)
Arterial hypertension, n (%)	3 (42.9)	1 (12.5)	8 (57.1)
Coronary heart disease, n (%)	0 (0)	1 (12.5)	1 (7)
Diabetes mellitus, n (%)	1 (14.3)	0 (0)	0 (0)
Echocardiography			
Maximal LVWT, mm	25.71 ± 5.06	22.00 ± 5.16	23.32 ± 6.32
Left atrial diameter, mm	46.86 ± 10.85	43.88 ± 5.17	43.64 ± 3.82
LA ESV index, ml/m2	43.09 ± 12.37	44.85 ± 10.76	44.84 ± 10.88
LV EDD index, mm/m2	22.96 ± 2.39	23.79 ± 2.61	23.77 ± 2.13
LV ESD index, mm/m2	10.04 ± 4.68*	13.95 ± 2.45	14.19 ± 2.83*
LV EDV index, ml/m2	46.16 ± 11.71	41.60 ± 11.94	45.22 ± 13.12
LV ESV index, ml/m2	12.60 ± 4.83	14.15 ± 5.65	15.61 ± 7.20
LVOT obstruction, n (%)	2 (28.5)	2 (25)	5 (35.7)
LVEF (%)	71.86 ± 4.88	65.75 ± 7.70	66.93 ± 7.00
E-e’ ratio	9.15 ± 4.91	9.43 ± 4.18	12.91 ± 7.95
Apical form of HCM, n (%)	1 (14.3)	1 (12.5)	3 (21.4)
Electrocardiography			
Pathological Q Waves, n (%)	1 (14.3)	3 (37.5)	3 (21.4)
T-Wave inversion, n (%)	5 (71.4)	5 (62.5)	12 (85.7)

Sokolow-Lyon index, mm	35.86 ± 23.66	30.00 ± 7.91	39.36 ± 18.20

P/LP variants - pathogenic/likely pathogenic variants. *Patients with P/LP variants in *MYH7* gene differ significantly from the combined group of patients including both carriers of P/LP variants in HCM-associated genes except MYH7 and MYBPC3 and genotype-negative HCM patients by LV ESD index according to Mann-Whitney test (p=0.037). BMI - body mass index, HCM - hypertrophic cardiomyopathy, SCD - sudden cardiac death, LVWT - left ventricular wall thickness, LA - left atrial; ESV - end-systolic volume, EDD - end diastolic diameter, ESD - end-systolic diameter, EDV - end-diastolic volume, ESV - end-systolic volume, LVOT - left ventricular outflow tract; LVEF - left ventricular ejection fraction.
